# PLGA-PEG Nanoparticles Coated with Anti-CD45RO and Loaded with HDAC Plus Protease Inhibitors Activate Latent HIV and Inhibit Viral Spread

**DOI:** 10.1186/s11671-015-1112-z

**Published:** 2015-10-22

**Authors:** Xiaolong Tang, Yong Liang, Xinkuang Liu, Shuping Zhou, Liang Liu, Fujina Zhang, Chunmei Xie, Shuyu Cai, Jia Wei, Yongqiang Zhu, Wei Hou

**Affiliations:** Huainan First People’s Hospital and First Affiliated Hospital of Medical College, Anhui University of Science and Technology, Huainan, 232001 China; The State Key Laboratory of Virology, Life Sciences College, Wuhan University, Wuhan, 430072 China; Clinical Laboratory, The Affiliated Huai’an Hospital of Xuzhou Medical College, Huai’an, 223002 China; School of Biotechnology, Southern Medical University, Guangzhou, 510515 China; Department of Medical Genetics, Tongji Medical College, Huazhong University of Science and Technology, Wuhan, Hubei 430030 People’s Republic of China

**Keywords:** HIV-1, CD45RO, Memory T cells, Nanoparticles

## Abstract

Activating HIV-1 proviruses in latent reservoirs combined with inhibiting viral spread might be an effective anti-HIV therapeutic strategy. Active specific delivery of therapeutic drugs into cells harboring latent HIV, without the use of viral vectors, is a critical challenge to this objective. In this study, nanoparticles of poly(lactic-co-glycolic acid)-polyethylene glycol diblock copolymers conjugated with anti-CD45RO antibody and loaded with the histone deacetylase inhibitor suberoylanilide hydroxamic acid (SAHA) and/or protease inhibitor nelfinavir (Nel) were tested for activity against latent virus in vitro. Nanoparticles loaded with SAHA, Nel, and SAHA + Nel were characterized in terms of size, surface morphology, zeta potential, entrapment efficiency, drug release, and toxicity to ACH-2 cells. We show that SAHA- and SAHA + Nel-loaded nanoparticles can target latently infected CD4^+^ T-cells and stimulate virus production. Moreover, nanoparticles loaded with SAHA + NEL were capable of both activating latent virus and inhibiting viral spread. Taken together, these data demonstrate the potential of this novel reagent for targeting and eliminating latent HIV reservoirs.

## Background

HIV infection is associated with a very high viral load in the body leading to a progressive depletion of immune cells, particularly CD4^+^ T cells [1, 2]. The infection can be treated with highly active antiretroviral therapy (HAART) consisting of at least three drugs from at least two classes of antiretroviral agents [3]. However, HAART cannot eliminate latent HIV reservoirs in resting CD_4_^+^/CD_45_RO^+^ T memory (Tm) cells [3, 4, 6]. The best way to completely eliminate this reservoir is to reactivate latent HIV-1 by inducing proviral gene expression [5]. Gene expression is controlled partly by the coiling and uncoiling of DNA around histones. This is accomplished with the assistance of histone acetyl transferases (HAT), which acetylate the lysine residues in core histone, resulting in less compact and more transcriptionally active chromatin [7, 8]. Gene expression from the HIV long terminal repeat can be modulated in this way to regulate latency. The histone deacetylase (HDAC) [9, 10] removes acetyl groups from lysine residues leading to the formation of condensed chromatin via a strong association between histones and the long terminal repeat of HIV, which obstructs the access of transcription factors and represses gene expression [9, 10].

HDAC inhibitors (HDACi) such as vorinostat, also known as suberoylanilide hydroxamic acid (SAHA), flush HIV out from reservoirs of latently infected cells by activating gene expression in proviruses [11, 12]. Concurrent treatment with an antiretroviral such as the protease inhibitor nelfinavir (Nel) and an HDACi simultaneously activates latent viruses and inhibits their spread. This treatment could produce a synergistic effect resulting in the elimination of latent HIV reservoirs [13, 14]. Efficient drug delivery by polymer-drug conjugates is a rapidly growing field. Advantages of the conjugates over treatment with drugs alone, demonstrated by clinical trials, include fewer side effects, enhanced therapeutic efficacy, and improved patient compliance. One of the most effective materials for this purpose is poly (lactic-co-glycolic acid)-polyethylene glycol (PLGA-PEG), a biodegradable polymer [15, 16]. Nanoparticles (NPs) of PLGA-PEG are formed by spontaneous association with amphiphilic surfactants in aqueous medium to form core (hydrophobic)-shell (hydrophilic) structures. Drugs can be loaded inside PLGA-PEG NPs regardless of their hydrophobicity, and sustained release of the drug can be maintained [15, 16]. However, the NPs’ limited targeting ability leads to the release of the loaded drug before reaching the site of action. Targeting can be facilitated by modification with a variety of functional molecules, including chemical compounds or antibodies, via carboxyl and or amine groups on the NP surface.

HIV mainly hides in “reservoirs” of CD4^+^ Tm cells [17, 18] which express the cell-specific surface antigen CD_45_RO. Specific binding to CD_45_RO through the single-chain variable fragment (scFv) CD_45_RO antibody can be used for targeted delivery of active drugs to human Tm cells [17–19], resulting in reduced toxicity and increased therapeutic effects of systemically delivered drugs. We designed a novel anti-HIV drug to specifically deliver SAHA and Nel to human Tm cells using scFv CD_45_RO-modified SAHA/Nel-loaded PLGA-PEG NPs (scFvCD_45_RO-SAHA/Nel-NPs). In this study, we used ACH-2 cells (HIV-1 latently infected cell lines) to determine the efficiency of activation of latent virus and inhibition of viral spread by scFvCD_45_RO-SAHA/Nel-NPs.

## Materials and Methods

### Materials

1-Ethyl-3-(3-dimethylaminopropyl)-carbodiimide (EDC), stannous octoate [Sn (Oct) 2: stannous 2-ethylhexanoate], n-hydroxysuccinimide (NHS), dicyclohexylcarbodiimde (DCC), and dimethyl sulfoxide (DMSO) were obtained from Sigma-Aldrich (St. Louis, MO, USA). All the other chemicals and reagents used were of analytical purity grade or higher. RPMI 1640 medium was obtained from Gibco Invitrogen (Carlsbad, CA, USA). Polyvinyl alcohol 205 (PVA 205; 88 % degree of hydrolyzation) was purchased from Kuraray Co., Ltd., China. 4′,6′-Diamidino-2-phenylindole (DAPI) was purchased from Molecular Probes (Eugene, OR, USA). The PLGA (*M*_w_ = 8.0 kDa; lactic acid: glycolic acid = 50:50) and PEG copolymer were purchased from Boehringer Ingelheim (Ingelheim am Rhein, Germany). Dimethylformamide (DMF) and acetonitrile are from Sigma-Aldrich. Human T lymphocyte (H9) and T lymphocytoid (ACH-2) cell lines, carrying one or two copies of integrated HIV-1 proviruses, were from the Experimental Animal Center (Wuhan University/ABSL-3 Laboratory, Wuhan, China).

### Cell Culture and HIV-1 Activation

H9 and ACH-2 cells were cultured in RPMI 1640 medium supplemented by 8–10 % fetal calf serum (HyClone). Activation of latent HIV-1 was evaluated with assays for HIV-1 RNA and p24 production. The amount of p24 was measured by use of HIV-1 p24 ELISA (Abnova, Walnut, CA, USA).

### Synthesis of PLGA-PEG Copolymer

PLGA-PEG diblock copolymers were synthesized by conjugating NH_2_-PEG-OH to PLGA-COOH via activation of carboxylic acid by NHS and EDC according to a described method [20, 21]: PLGA-COOH (3.6 g, 0.2 mmol) was converted to PLGA-NHS with excess NHS (95 mg, 0.8 mmol) in EDC (150 mg, 0.8 mmol) and methylene chloride (7.0 mL) while stirring over for 24 h. Unreacted NHS and EDC were removed by the use of a solution containing 70 % ethyl ether and 30 % methanol. After precipitation with ethyl ether, PLGA-NHS was dissolved in chloroform, followed by addition of NH_2_-PEG-OH (0.05 mmol) and n,n-diisopropylethylamine (Fisher Scientific) (0.2 mmol) and stirred overnight. The resulting PLGA-PEG copolymers were precipitated with cold methanol and dried under vacuum for storage prior to further treatment.

### Preparation of Antibody-Coated and Drug-Loaded PLGA-PEG NPs

For scFv CD_45_RO antibody conjugation to PLGA-PEG, the PLGA-PEG (10 mg) and antibody (2.5 mg) were dissolved in 600 μL of 50/50 acetonitrile/DMF [22, 23] and stirred overnight. The product was precipitated with 2 mL of an ice-cold 80/20 mix of diethyl ether/methanol, centrifuged at 4500*g* for 15 min, and re-dissolved in 600 μL 50/50 acetonitrile/DMF. This cycle was repeated three times, and the antibody-PLGA-PEG polymer was dried under vacuum. Antibody-PLGA-PEG NPs, simultaneously loaded with SAHA and Nel, were prepared via a single oil/water emulsion and evaporation method [24, 25]. One hundred milligrams of antibody-PLGA-PEG NPs, 25 mM of SAHA, and 25 mM of Nel were dissolved in 1.5 ml of dichloromethane, which formed the organic phase. The organic phase was emulsified with 4 ml of pH 7.4 phosphate-buffered saline containing PVA 205 (3 %, *w*/*v*) by probe sonication (VC 505, Vibra-Cell Sonics, Newtown, CT, USA) at 45 W for 30 s in an ice bath. The organic suspension was stirred for 5 h at 25 °C and evaporated completely under reduced pressure. The NPs were then isolated by ultracentrifugation (25,000 rpm for 10 min), washed with ddH_2_O, and lyophilized. NPs containing only SAHA or Nel were prepared by the use of the aforementioned oil/water emulsion method [24, 25]. Morphological evaluation, average size, and zeta potential of the NPs were analyzed by the use of transmission electron microscope (TEM; JEOL JEM 1200 EXII) and a dynamic light-scattering detector (DLS; Malvern Zetasizer 2000, Malvern, UK). In order to obtain fluorescing images, NPs were loaded with 1 % (*w*/*w*) coumarin-6.

### Measurement of Drug Loading, Entrapping, and Release

To determine the amount of drug loaded, single or dual drug-loaded NPs (5.0 mg) were dissolved in 1.5 mL of dimethylsulfoxide for 15 min by sonication. Then, 2.0 mL of methanol was added to precipitate the dipolymer. After centrifugation at 1500*g* for 20 min, the drug quantity in the supernatant was analyzed by high-performance liquid chromatography (HPLC). The loading content (LC) and entrapment efficiency (EE) of the drug-loaded NPs were calculated by the following equations [26, 27]:

$$ \mathrm{L}\mathrm{C}\kern0.5em \%=\left(\mathrm{weight}\ \mathrm{of}\kern0.5em \mathrm{drug}\kern0.5em \mathrm{in}\ \mathrm{the}\ \mathrm{N}\mathrm{P}\mathrm{s}\right)/\left(\mathrm{weight}\ \mathrm{of}\ \mathrm{the}\ \mathrm{N}\mathrm{P}\mathrm{s}\right) \times 100\ \%; $$

$$ \mathrm{E}\mathrm{E}\kern0.5em \%=\left(\mathrm{weight}\ \mathrm{of}\kern0.5em \mathrm{drug}\kern0.5em \mathrm{in}\ \mathrm{the}\ \mathrm{N}\mathrm{P}\mathrm{s}\right)/\left(\mathrm{input}\kern0.5em \mathrm{weight}\ \mathrm{of}\kern0.5em \mathrm{drug}\right) \times 100\ \% $$

To measure drug release from NPs, single or dual drug-loaded NPs were lyophilized, weighed, re-suspended in PBS/0.1 % Tween-80 at pH 7.0, and incubated in a vibrating water bath at 37 °C and 130 rpm. At various times between 30 min and 10 days, samples were taken out and centrifuged at 25,000 rpm for 15 min. An aliquot of the supernatant was removed for quantification; this volume was replaced with fresh PBS/0.1 % Tween-80 at pH 7.0. Drug release was estimated with HPLC. The cumulative release of drug was plotted against time.

### Cellular Uptake and Intracellular Localization of NPs

To quantitate uptake kinetics, coumarin-6 NPs were prepared for observation with confocal microscopy. T cells were seeded at 1 × 10^3^ cells per well and incubated with coumarin-6 NPs suspended in medium with 5 μM rhodamine at 37 °C with 5 % CO_2_. At various time points (from 30 min to 6 h), wells were treated with Hoechst 33342 nucleic acid stain (Invitrogen) for 15 min. The medium was removed, and cells were washed three times with PBS and fixed with methanol for 25 min. Confocal fluorescence images were acquired with a Nikon Ti-E microscope equipped with an UltraVIEW VoX confocal attachment (Perkin Elmer).

### Cytotoxicity Assays

The cytotoxicity of NPs was evaluated in ACH-2 cells by the use of the Cell-Quant™ alamarBlue cell viability reagent (GeneCopoeia, Rockville, MD, USA). Briefly, cells were seeded in 96-well plates (Costar, Chicago, IL, USA) at 5 × 10^2^ viable cells/well in Dulbecco’s modified Eagle’s medium (Invitrogen, USA) supplemented with 10 % heat-inactivated fetal bovine serum (HyClone, USA) under 5 % CO_2_ at 37 °C and incubated for 24 h to allow cell attachment. The medium was replaced with 150 μL of medium containing NPs at various concentrations (0.1 to 32 mg/mL) and incubated for 48 h. Then, 20 μL of the alamarBlue cell viability reagent was added to the culture medium for 4 h. Fluorescence was measured at *E*_x_/*E*_m_ = 545/590 nm. For cell viability, 400 μL of cell suspension (1 × 10^4^ cells/mL) was mixed with 0.1 mL of 0.4 % trypan blue dye, after being incubated at 25 °C for 2 min. The cell viability rate was calculated as followed: cell viability (%) = (number viable cells/number total cells) × 100 %.

### Protein Extraction and Western Blot Analysis

Cells (2.0 × 10^6^) with different treatments were collected and centrifuged at 1500 rpm for 15 min at 4 °C and then washed with PBS. Protein was extracted from cells of different groups with Triton X100 lysis buffer (100 μl) and quantified by the use of the BCA Protein Assay (Pierce Biotechnology). Proteins were separated on gradient SDS-PAGE gels and transferred to nitrocellulose membranes. After blocking in 5 % (*w*/*v*) skim milk at room temperature for 30 min, membranes were incubated with the specific primary antibody overnight at 4 °C. Following incubation with secondary antibody conjugated to horseradish peroxidase for 1 h at room temperature, the bands were analyzed with the enhanced chemiluminescence system (Pierce Biotechnology).

### HIV p24 Protein Level

HIV-1 p24 antigen was quantified in drug-treated cell culture supernatants by the use of a commercially available ELISA kit (p24 HIV antigen ELISA kit, Perkin Elmer) according to the manufacturer’s protocol.

### Quantification of HIV RNA Transcripts

Total RNA was extracted from ACH-2 cells or culture supernatants by TRIzol. HIV RNA levels were measured by RT-qPCR (Roche TaqMan 2.0). The following primer sets were used for PCR amplification: histone deacetylase 4 (HDAC4) mRNA forward 5′-GGTTTATTCTGATTGAGAACTGG-3′ and reverse 5′-ATTGTAAACCACAGTGCTCGC-3′; HIV Gag mRNA forward 5′-GGTGCGAGAGCGTCAGTATTAAG-3′ and reverse 5′-AGCTCCCTGCTTGCCCATA-3′; and GAPDH mRNA forward 5′-CGGAGTCAACGGATTTGGTCGTAT-3′ and reverse 5′-AGCCTTCTCCATGGTGGTGAAGAC-3′.

### Statistical Analysis

Results were expressed as mean ± standard error of the mean (SEM). The statistical significance between groups was determined using either unpaired or paired tests. *p* values of <0.05 were considered statistically significant.

## Discussion

### Characterization of the Antibody-PLGA-PEG Copolymer

**Fig. 1:**
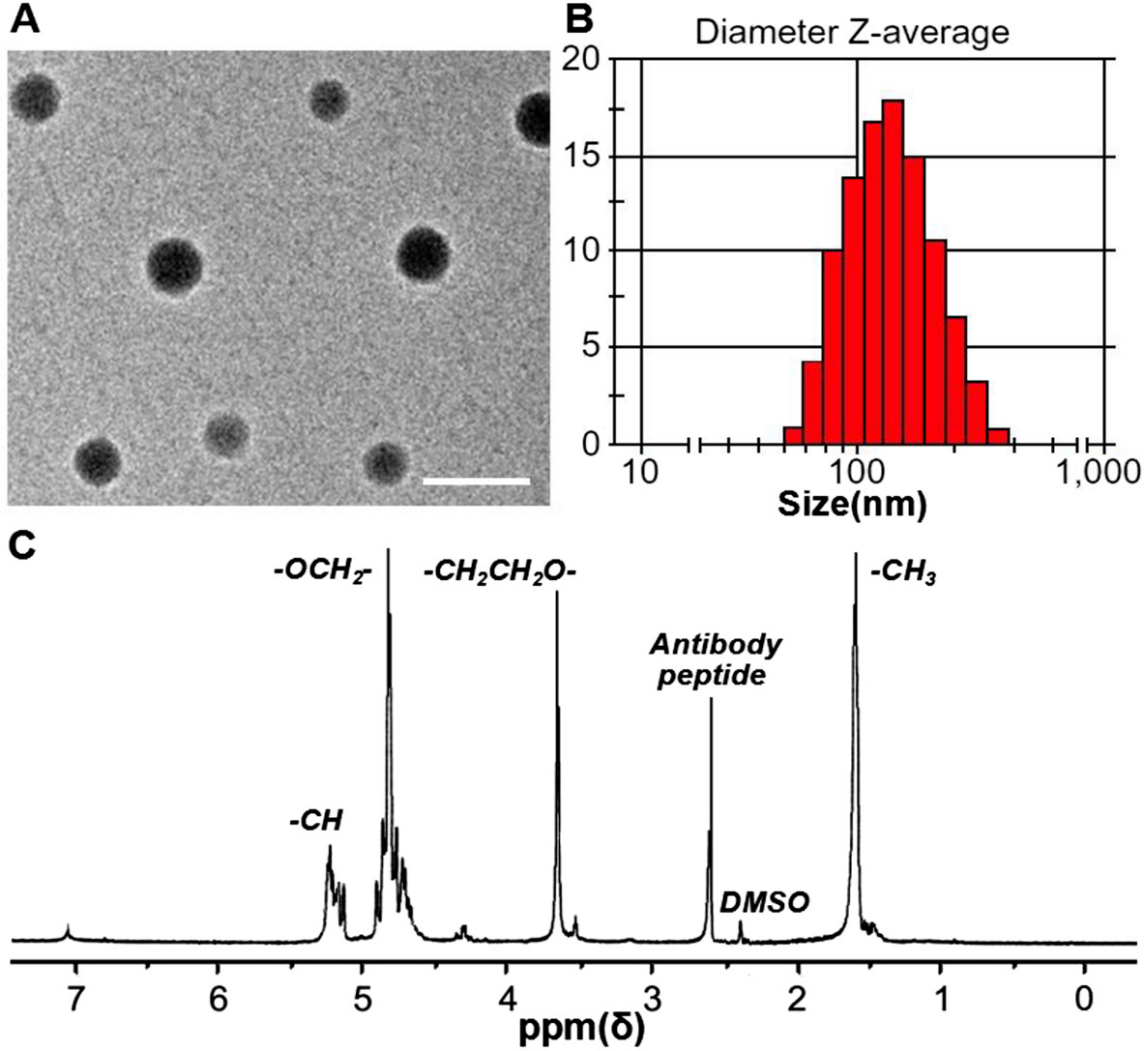
Characterization of nanoparticles. **a** TEM images of the representative antibody-PLGA-PEG NPs. The scale bar is 200 nm. **b** Diameter *Z*-average of antibody-PLGA-PEG NPs. **c** A representative ^1^H-NMR spectrum of the antibody-PLGA-PEG copolymer

Initial analysis of NP morphology by TEM (Fig. 1a) revealed a spherical shape, a particle size of about 125 nm, and a narrow size distribution: an excellent range for tumor penetration and retention [28]. Dynamic light scattering analysis confirmed the TEM data on NP size (Fig. 1b). Compared with Ab-SAHA and Ab-Nel NPs, which have a ζ potential value of −16.5 and −16.2 mV, respectively, the Ab-SAHA/Nel NPs exhibited higher ζ potential values of around −14.6 mV. The size variation of synthetic NPs had no significant effect on the ζ potential values (Table 1). The negative surface charge may be due to the presence of ionized carboxyl groups on PLGA segments [29]. The antibody PLGA-PEG copolymer was dissolved in DMSO and analyzed with ^1^H-NMR spectroscopy. The ^1^H-NMR peaks were as follows: the characteristic peak of the -CH_2_ (ethylene glycol protons) was at 3.6 ppm and the peaks of the -CH (lactide proton), -CH_2_ (glycolide proton), and -CH_3_(lactide proton) were at 5.2, 4.8, and 1.7 ppm, respectively (Fig. 1c). These ^1^H-NMR spectra confirmed peptide coupling to PLGA-PEG copolymer as well as the presence of both PLGA and PEG domains in the PLGA-PEG synthetic copolymer.

**Table 1:** Physicochemical characteristics of synthetic NP formulations

Formulation	Size (nm)	PDI	ZP (mV)	EE (%)
Ab-SAHA NPs	119 ± 3.2	0.13 ± 0.005	−16.5 ± 1.7	80
Ab-Nel NPs	118 ± 2.3	0.14 ± 0.006	−16.2 ± 1.5	84
Ab-SAHA/Nel NPs	125 ± 1.9	0.17 ± 0.008	−14.6 ± 4.3	78^a^
				80^b^

*PDI* polydispersity index, *ZP* zeta potential (mV), *EE* encapsulation efficiency (%)

^a^SAHA

^b^Nel

### Drug Release Profiles

**Fig. 2:**
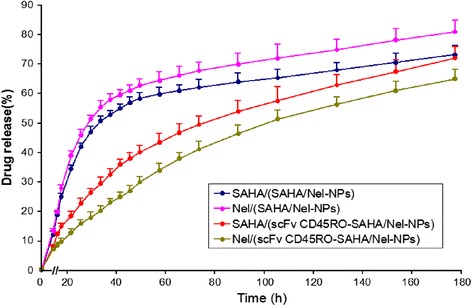
In vitro release profiles of SAHA and Nel from NPs incubated in phosphate-buffered saline (pH 7.0). The data are presented as mean ± SEM (*n* = 3)

Both SAHA and Nel encapsulated into NPs in a 1:1 molar ratio. As shown in Fig. 2, the non-Ab-modified nanoparticles showed biphasic release characteristics, i.e., an immediate release (“burst effect”) followed by a slower release profile. However, no initial burst release was observed for Ab-modified NPs. The drugs (SAHA and Nel) were released from Ab-modified nanoparticles in a sustained manner for up to 180 h, providing a prolonged biological effect. On this basis, these NPs were selected for further biological studies.

### Ex Vivo Study of scFv CD_45_RO-Coumarin-6 NP Cellular Uptake

**Fig. 3:**
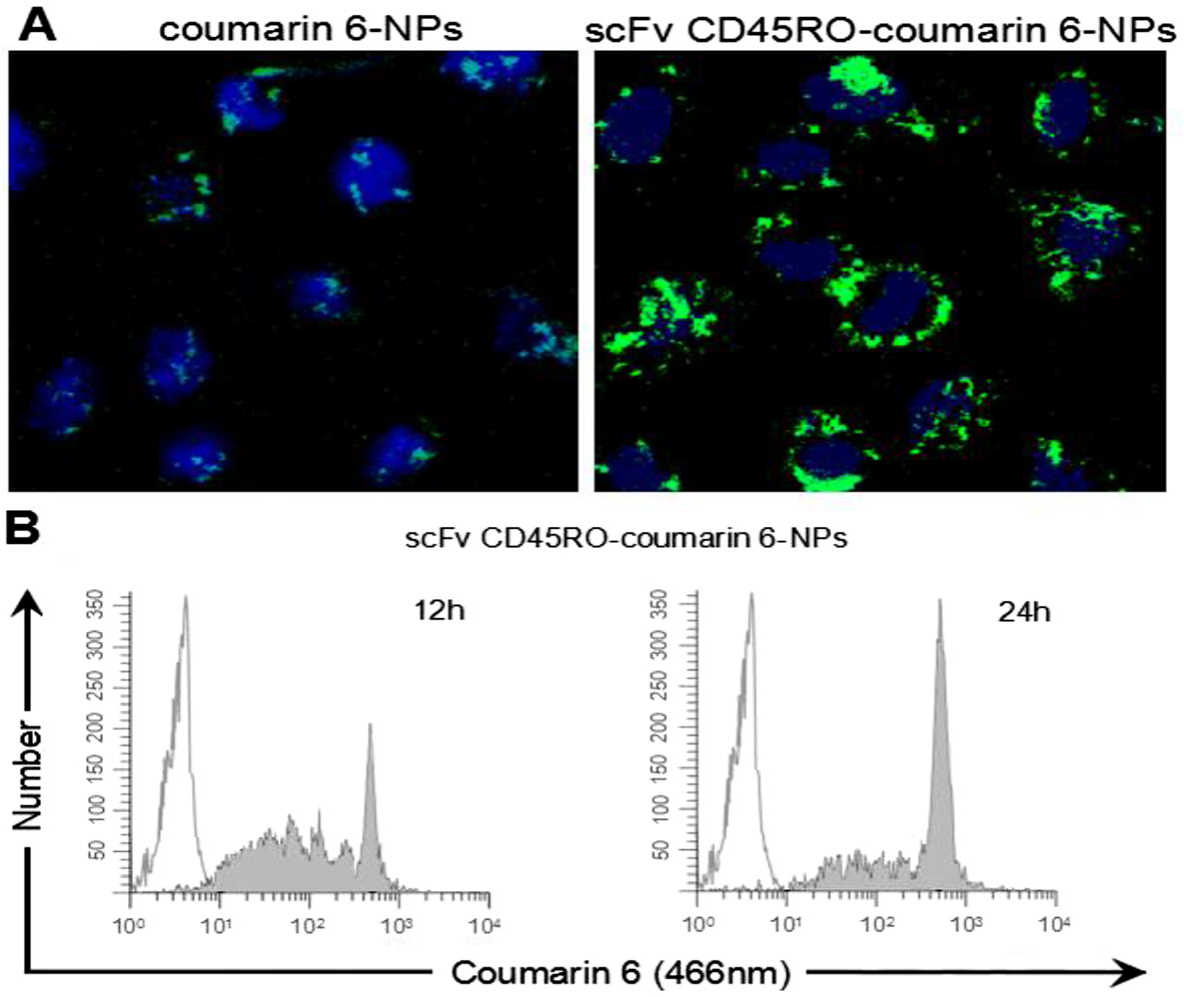
Cellular uptake analysis of coumarin-6-labelled NPs. **a** Confocal images of H9 cells. Cells were treated with scFv CD_45_RO-coumarin-6 NPs or coumarin-6 NPs and incubated for 12 h. Nuclei were stained with Hoechst 33342 (*blue*) and coumarin-6 (*green*) (×400). **b** Flow cytometry analysis of cellular uptake of scFv CD_45_RO-coumarin-6 NPs in a time-dependent manner. Representative histogram overlay (*white peaks,* 0 h post incubation; *gray peaks*, 12 h post incubation)

In order to evaluate our NPs as delivery vehicles for target cells, we loaded them with the fluorescent dye coumarin-6 and incubated them with H9 cells for 12 h, and the uptake was evaluated by flow cytometry and confocal laser scanning microscope (CLSM). As shown in Fig. 3, scFv CD_45_RO-coumarin-6 NPs showed a definite cellular uptake within 12 h. In contrast, the coumarin-6 NPs showed little uptake during the same incubation period. The merged images show cytosolic localization of scFv CD_45_RO-coumarin-6 NPs that is distinct from the nuclear Hoechst 33342 stain. These results demonstrate the time-dependent cellular internalization of scFv CD_45_RO-coumarin-6 NPs. It is likely that the analogous NPs loaded with drugs will show a similar trafficking pattern and release their cargo after internalization, reducing the non-specific distribution and increasing the intracellular concentrations of the drugs. This effect would, in turn, enhance drug interactions with HIV components and improve their therapeutic effects.

### Cytotoxic Effects of NPs on ACH-2 Cells

**Fig. 4:**
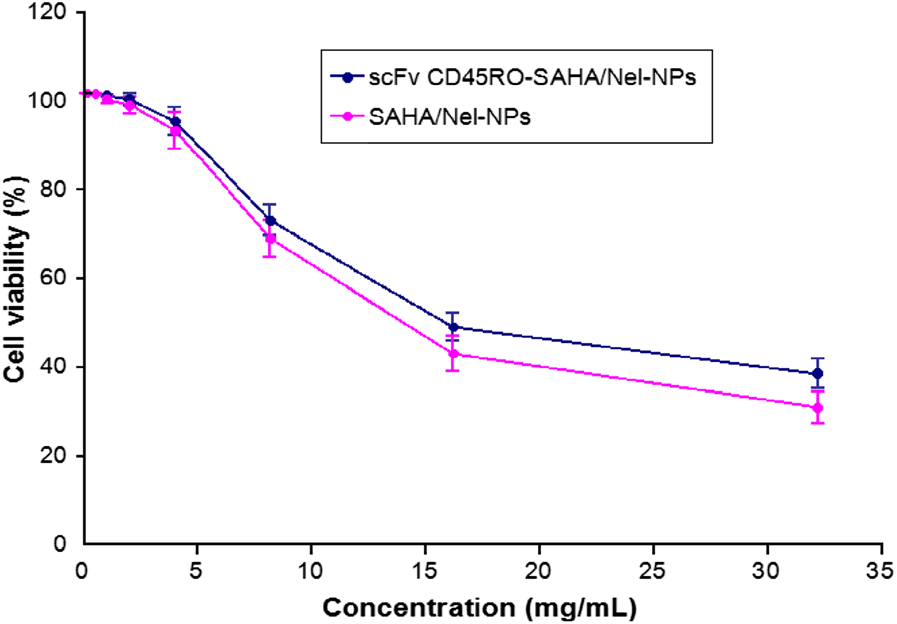
Cytotoxicity of drug-loaded NPs in ACH-2 cells with the Cell-Quant™ alamarBlue cell viability reagent assay. The data were presented as mean ± SEM (*n* = 3)

To assess the cellular cytotoxicity of the NPs, ACH-2 cells were incubated with NPs (0.1 to 32 mg/mL) and analyzed for cell viability. After 48 h of exposure to the NPs, cell viability was assessed using the Cell-Quant™ alamarBlue cell viability reagent (GeneCopoeia, Rockville, MD, USA). The results of three independent assays were plotted onto a fitted curve to obtain median lethal dose (LD_50_) values. The experimental LD_50_ values were 13.8 mg/mL for SAHA/Nel NPs and 16.0 mg/mL for scFv CD_45_RO-SAHA/Nel NPs. The experimental LD_50_ for scFv CD_45_RO-SAHA/Nel NPs corresponds to approximately 5–10 g/kg of body weight, which is much higher than the intravenous dose required for in vivo drug delivery (Fig. 4). These results suggest that the in vitro cytotoxicity of the scFv CD_45_RO-SAHA/Nel NPs was low.

### scFv CD_45_RO-SAHA/Nel NPs Activate Latent HIV in ACH-2 Cells

**Fig. 5:**
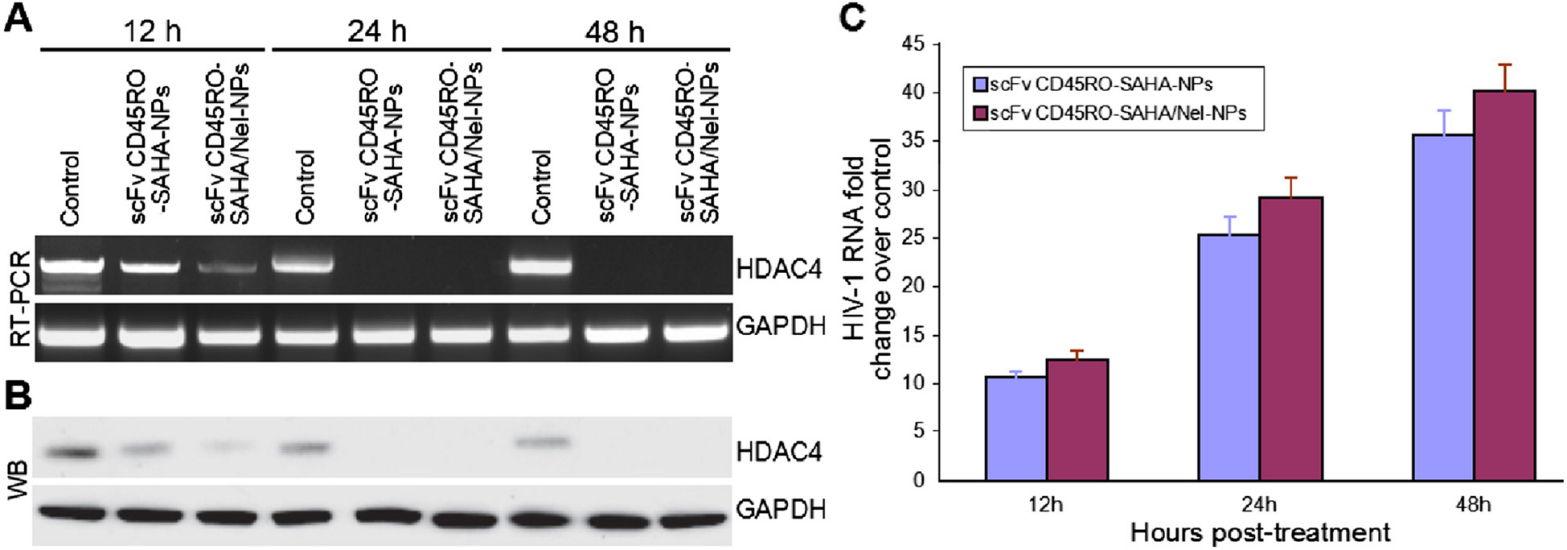
HDAC4 in ACH-2 cells treated with scFv CD_45_RO-SAHA NPs or scFv CD_45_RO-SAHA/Nel NPs was downregulated, leading to HIV-1 reactivation in ACH-2 cells. **a** Real-time PCR quantification of HDAC4 mRNA expression and **b** immunoblotting of HDAC4 protein levels on whole-cell lysates from ACH-2 cells cultivated in the presence scFv CD_45_RO-SAHA NPs, scFv CD_45_RO-SAHA/Nel NPs, or control (DMEM) at the indicated times. **c** Real-time PCR quantification of HIV-1 RNA in ACH-2 cells treated with PBS (control), scFv CD_45_RO-SAHA NPs, or scFv CD_45_RO-SAHA/Nel NPs for the indicated times. Results were expressed as the fold change vs. the control at each time point (mean ± SEM of three independent experiments)

SAHA can induce hyperacetylation of the HIV promoter-associated nucleosomes to activate latent HIV by suppressing cellular HDAC expression and binding to the HIV promoter [30–32]. We investigated the change in HDAC4 expression in ACH-2 cells incubated with scFv CD_45_RO-SAHA/Nel NPs and scFv CD_45_RO-SAHA NPs. Our results showed a gradual reduction in HDAC4 mRNA and protein expression in cells treated with both scFv CD_45_RO-SAHA/Nel NPs and scFv CD_45_RO-SAHA NPs, reaching virtually undetectable levels after 24 h. (Fig. 5a, b). At the same time, viral RNA levels progressively increased, reaching about 40-fold higher levels than the control after 48 h of incubation (Fig. 5c). Therefore, scFv CD_45_RO-SAHA/Nel NPs stimulated HIV-1 mRNA expression and inhibited cellular HDAC4 expression.

### scFv CD45RO-SAHA/Nel NPs Inhibit HIV Infection

**Fig. 6:**
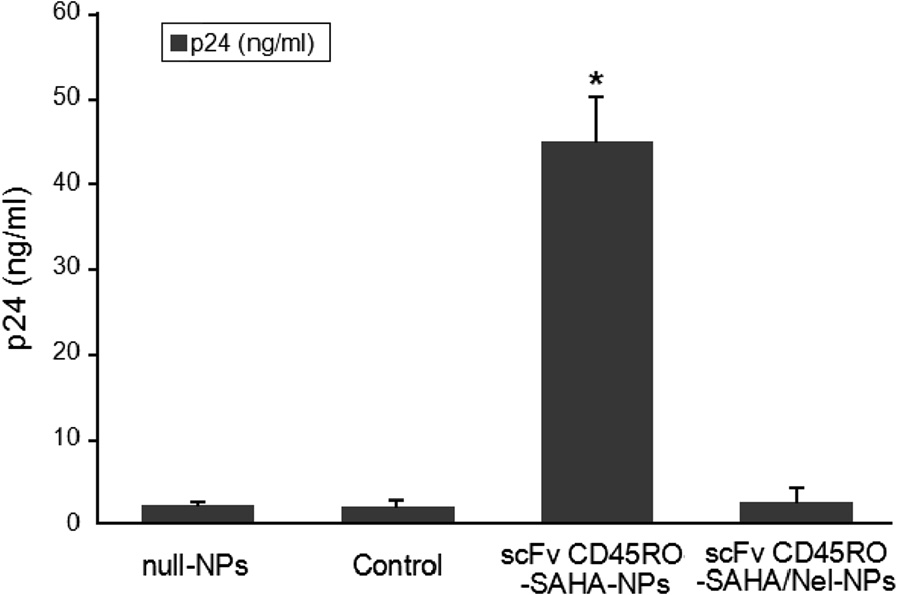
Simultaneous incorporation of suberoylanilide hydroxamic acid (SAHA) and protease inhibitor nelfinavir (Nel) in scFv CD_45_RO-SAHA/Nel NPs can both activate latent HIV expression and inhibit viral spread. ACH-2 cells were incubated for 3 days in the presence of the indicated drug combinations delivered by NPs. Conditioned medium was transferred to naïve cells, and viral p24 protein in the culture supernatant was measured by ELISA. *Error bars* indicate the standard deviation of triplicate data points. *Asterisk* means significant difference as compared with the rest of the groups at *P* < 0.05

In order to analyze the efficacy of the scFv CD_45_RO-SAHA/Nel NPs to activate latent proviruses [33, 34], ACH-2 cells were incubated in the presence of 2.0 mg/mL scFv CD_45_RO-SAHA/Nel NPs, scFv CD_45_RO-SAHA NPs, or control (DMEM) for 24 h at 37 °C. The conditioned medium from these cells was then transferred to untreated cells and incubated for 72 h. At this time, HIV p24 could hardly be detected in the scFv CD_45_RO-SAHA/Nel-NP-treated cells, while in scFv CD_45_RO-SAHA-NP-treated cells, p24 levels were significantly elevated relative to the control. These data confirmed that scFv CD_45_RO-SAHA/Nel NPs could inhibit virus spread in ACH-2 cells (Fig. 6). Conceptually, this finding represents a safety feature whereby any virus induced by the latency activator is also inactivated by the protease inhibitor, thereby impeding virus spread. In this way, the limited production of viable virus would facilitate subsequent targeting by the immune response. Hence, the scFv CD_45_RO-SAHA/Nel NPs can effectively stimulate latent virus and also inhibit virus spread, which further exemplifies the potential benefits of using nanoparticles in HIV purging strategies.

## Conclusion

HIV-1 mainly targets human CD_4_^+^ T cells that continue to harbor latent proviruses after infection. Eradicating latent HIV in Tm cellular reservoirs is the most important barrier to curing AIDS [34]. SAHA is a potent HDAC inhibitor capable of reactivating HIV-1 expression from latently infected cells [9–11], and Nel is a potent bioavailable HIV-1 protease inhibitor, which is widely prescribed in combination with HIV reverse transcriptase inhibitors for the treatment of HIV infection. We have developed NPs, conjugated to the Tm cell-specific antibody scFv CD_45_RO, which are able to specifically bind to Tm cells. When high concentrations of SAHA were delivered from these particles, a stable increase in viral reactivation without detectable cytotoxicity occurred. Moreover, scFv CD_45_RO-conjugated NPs loaded with both SAHA and Nel produced particles capable of both activating latent virus and inhibiting viral spread. Taken together, these data demonstrate the ability of nanotechnology to improve conventional approaches for activating latent HIV. This study provides a key proof-of-principle experiment, which opens up avenues to eradicate HIV infection by eliminating latent viral reservoirs via T-cell-specific delivery of an HDAC inhibitor in combination with a protease inhibitor.
